# Hoch dosierte Anderson- und Kestenbaum-Operation bei Nystagmus mit Kopfzwangshaltung

**DOI:** 10.1007/s00347-020-01086-6

**Published:** 2020-03-26

**Authors:** Michael Gräf, Anja Hausmann, Dominik Kowanz, Birgit Lorenz

**Affiliations:** 1 Fachbereich Medizin, Justus-Liebig-Universität Gießen, Gießen, Deutschland; 2 Klinik und Poliklinik für Augenheilkunde, Universitätsklinikum Gießen und Marburg GmbH, Standort Gießen, Friedrichstr. 18, 35392 Gießen, Deutschland

**Keywords:** Augenmuskelchirurgie, Kopfschiefhaltung, Neutralzone, Torticollis, Umlagerungsoperation, Eye musle surgery, Null zone shift, Neutral zone, Torticollis, Extraocular muscle surgery

## Abstract

**Hintergrund:**

Die Korrektur einer Kopfzwangshaltung (KZH) bei infantilem Nystagmussyndrom (INS) erfolgt mit der Kestenbaum-Operation (KO) in Form der beidseitigen Rücklagerung der in KZH aktiven Agonisten und Verkürzung ihrer Antagonisten oder mit der Anderson-Operation (AO) durch die alleinige Rücklagerung der Agonisten.

**Ziel der Arbeit:**

Vergleich der Ergebnisse hoch dosierter AO und KO bei ähnlicher KZH.

**Patienten und Methoden:**

In unterschiedlichen Zeiträumen (2013 bis 2019 bzw. 2003 bis 2013) kam ausschließlich die AO bzw. die KO zur Anwendung. Eine hoch dosierte AO erhielten in einer konsekutiven Fallserie 33 Orthotrope mit INS und KZH, eine KO erhielten 19 Patienten. Die Mediane und Streubreiten (min–max) in den Gruppen AO/KO betrugen: Alter bei OP 7 (4–44) Jahre/6 (4–27) Jahre; KZH 32,5°(20–45)/30°(17–40); Operationsstrecke pro Auge AO 13 (10–16) mm (Standarddosis), KO 10 (6–12) mm + 10 (6–12) mm (Mittel 0,60 mm/°KZH).

**Ergebnisse:**

Die KZH betrug nach ca. 3 Monaten 10°(−3–20)/10°(−7–20), bei der Spätkontrolle (8 bis 153 Monate) 10°(0–20)/10°(−27–30). Sie war bei der letzten Kontrolle um 67 % (20–100)/64 % (14–100) verringert. Eine Restdrehung ≤15° fand sich in 79/81 % der Fälle (91 % nach AO ≥13 mm; *n* = 23), ein Rest ≤10° in 55/57 %, (65 % nach AO ≥13 mm). Die Ad‑/Abduktionsfähigkeit der rückgelagerten Muskeln betrug nach AO 30°/30° (10–37/15–45), nach KO 32°/30° (10–40/12–45). Eine assoziierte Kopfneigung wurde durch den Eingriff nicht verbessert. Der mittlere Anstieg des binokularen Visus betrug jeweils <1 Zeile, in der Gruppe AO 1 Zeile bei Kindern ≤6 Jahre, kein Anstieg in der Altersklasse >6 Jahre.

**Schlussfolgerung:**

AO und KO waren bei der verwendeten Dosierung wirkungsgleich. Die geringere Invasivität der AO spricht für deren Anwendung als Ersteingriff.

## Abstract (en)

**Background:**

The correction of anomalous head posture associated with infantile nystagmus syndrome (INS) is carried out by the Kestenbaum procedure (KP) combining bilateral horizontal yoke muscle recession with resection of their antagonists or by the Anderson procedure (AP) which is confined to yoke muscle recession alone.

**Aim of the study:**

To compare the results of highly dosed AP and KP for anomalous head turn (HT).

**Patients and methods:**

The AP (2013–2019) and KP (2003–2013) were used as the exclusive procedures during the respective time periods. A highly dosed AP was performed in a case series of 33 consecutive orthotropic patients with INS and KP in 19 orthotropic patients with INS. Medians and ranges (min–max) in groups AP/KP were: age at surgery 7 years (4–44 years)/6 years (4–27 years), HT 32.5° (20–45°)/30° (17–40°) and surgery on each eye AP 13 mm (10–16 mm) (standard dose), KP 10 mm (6–12 mm) + 10 mm (6–12 mm) (mean 0.6 mm/°HT).

**Results:**

The HT was diminished to 10° (−3–20°)/10° (−7–20°) ca. 3 months after surgery. At the late visit (8–153 months), the residual HT was 10° (0–20°)/10° (−27–30°). The HT was reduced by 67% (20–100%)/64% (14–100%) at the last control. A residual HT ≤15° was present in 79%/81% of cases (91% after AP ≥13 mm; *n* = 23) and HT ≤10° in 55%/57%, (65% after AP ≥13 mm). The maximum adduction/abduction towards the recessed muscles was 30°/30° (10–37°/15–45°) after AP and 32°/30° (10–40°/12–45°) after KP. An associated head tilt was not improved by the intervention. Mean binocular visual acuity increased by <1 line in both procedures. After AP the mean increase was 1 line in children ≤6 years old and there was no increase in patients >6 years of age.

**Conclusion:**

The effectiveness and success rates of AP and KP were similar with the dosing used. Less invasive surgery is an advantage in favor of AP as the primary procedure.

Eine große nystagmusbedingte Kopfzwangshaltung (KZH) ist eine Indikation zur operativen Therapie. Die dabei häufigste KZH ist eine Kopfdrehung aufgrund einer seitlich gelegenen Nystagmusneutralzone. Wenn der Nystagmus beim Blick nach rechts abnimmt, wird der Patient den Kopf bei Visusanforderung nach links wenden. Im Blick noch weiter nach rechts, also rechts der Neutralzone, schlägt der Nystagmus nach rechts, links der Neutralzone nach links. Dieses typische Verhalten des idiopathischen infantilen (IIN) und des mit sensorischen Defekten assoziierten Nystagmus (SDN) ist unabhängig davon, mit welchem Auge der Patient fixiert, und unter monokularen und binokularen Sehbedingungen annähernd gleich. Die KZH kann zusätzlich vertikale oder Neigungskomponenten aufweisen. Davon zu unterscheiden ist der Nystagmus vom Latenstyp. Er ist nicht Gegenstand dieser Studie. Zur Korrektur der KZH dienen Umlagerungsoperationen. Sie verlagern die Neutralzone hin zur Primärposition [1, 14, 15]. Wenige Patienten sprechen auf das Prinzip artifizielle Divergenz (AD) an. AD erzeugt eine Exophorie, deren Kompensation durch Konvergenz den Nystagmus insgesamt dämpft [3]. AD allein genügt selten, ist aber mit Umlagerungsoperationen kombinierbar [24, 25, 30]. Die sog. 4‑Muskel-Tenotomie hat sich nicht durchgesetzt [9].

Die Protagonisten der Umlagerungsoperationen, Kestenbaum und Anderson, berichteten zeitgleich über die Anwendung ihrer prinzipiell ähnlichen Konzepte [1, 14]. Kestenbaum lagerte die in der KZH aktiven Agonisten beidseits zurück und resezierte die Antagonisten [14, 15]. Die Dosis von 1 mm pro Muskel je 5° Kopfdrehung (0,4 mm/Auge/°KZH) war nicht ausreichend. In der Folge wurden Dosierungen bis >0,6 mm/Auge/°KZH empfohlen, was >10 mm pro Muskel entsprechen kann [6, 12, 13, 16, 18, 20, 22, 27]. Auch damit wurde nicht selten eine Rest-KZH beobachtet. Anderson lagerte nur die Agonisten zurück [1]. Hierzu liegen relativ wenige Studien vor. Expertenmeinungen waren geteilt. Von Noorden favorisierte eine hoch dosierte AO als Ersteingriff [21]. De Decker machte die Wahl des Verfahrens von der Größe der KZH abhängig [4]. Buckley berichtete, dass reine Rücklagerungen, selbst hoch dosiert, auf Dauer meist ineffektiv wären (in [23]). Systematische Studien zur Anderson-Operation (AO) erschienen um die Jahrtausendwende. Darin wurde die AO als sehr effektiv beschrieben [2, 5, 11, 17, 19, 26]. In unserer Klinik lieferte die AO verglichen mit früheren Kestenbaum-Operationen (KO) nahezu identische Erfolgsraten nach 3 bis 6 Monaten [7]. Daher verwenden wir seit 2013 die AO zur Therapie bei nystagmusbedingter Kopfdrehung. Bei den Nachkontrollen fiel auf, dass die Rest-KZH in einem längeren Zeitintervall im Mittel etwas zunahm [8]. Frühere Untersuchungen zum Effekt der KO in unserer Klinik waren auf kurze Nachbeobachtungszeit begrenzt, und die Dosierung war niedriger als in den letzten Jahren [6]. Daher verglichen wir rezentere, höher dosierte KO und die hoch dosierte AO im Hinblick auf ihre Langzeitwirkung.

## Patienten und Methoden

### Patienten

Die von der Ethikkommission der Justus-Liebig-Universität Gießen genehmigte (PN 130/15) retrospektive Studie umfasst 47 Orthotrope, für die eine AD nicht infrage kam, die seit 2013 in unserer Klinik eine hoch dosierte AO zur Korrektur ihrer nystagmusbedingten Kopfdrehung erhielten. Nicht in die aktuelle Auswertung eingeschlossen wurden 3 Patienten, die bereits eine Augenmuskeloperation erhalten hatten, 4 Patienten, die nicht zur Spätkontrolle kamen, und 3 Patienten, die nach 4 Monaten einen Zweiteingriff erhielten (1 präoperativ kompensierte, postoperativ trotz asymmetrischer Rücklagerungen dekompensierte Exophorie, 1 Optimierung des guten OP-Erfolgs durch AD, 1 Verstärkung der AO durch bovines Perikard-Interponat). Auch 4 Patienten, deren Eingriff erst kurz zurücklag, wurden nicht in die Studie aufgenommen. Die Daten der verbleibenden 33 in den Jahren 2013 bis 2018 operierten Patienten wurden ausgewertet. Zum Vergleich wurden Daten von 19 Patienten ausgewertet, die in den Jahren 2003 bis 2013 eine KO und auch schon eine Spätkontrolle erhalten hatten oder zu diesem Zweck einbestellt wurden. Bei 14 Patienten waren die OP-Strecken seitengleich, bei 5 Patienten am adduzierten Auge maximal 2 mm größer als am abduzierten Auge. Ein Patient, dessen Kopfdrehung sich spät nach KO umkehrte (Tab. 2, Nr. 17), wurde bei der vergleichenden Analyse der Langzeitergebnisse ausgeschlossen.

### Diagnostik

Die Patienten erhielten regelmäßig eine Refraktometrie in Zykloplegie, Spaltlampenuntersuchung, Ophthalmoskopie in Mydriasis, Biometrie der axialen Bulbuslänge und wenn möglich ein Fundus-OCT. Die Refraktion war abzüglich 0,5 dpt sph korrigiert. Der Visus wurde monokular und binokular in KZH bestimmt, die Augenstellung im einseitigen und alternierenden Prismenabdecktest, das Binokularsehen im Bagolini‑, Titmus- und Lang-1-Stereotest untersucht. Die KZH (auch Komponenten in anderer Ebene) war vor und nach KO mit einem orthoptischen Goniometer (Strabofix) gemessen. Vor und nach AO wurde die Kopfdrehung in der Ferne zudem an einer 12 m breiten Tangentenskala bestimmt. Die Position an der Skala, aus der für den Untersucher beide Ohren des Patienten gleich sichtbar sind, entspricht der KZH. So sind evtl. Fehler, z. B. durch Verrutschen eines Stirnprojektors, ausgeschlossen. Im Zentrum der Skala wurden Optotypen angeboten. Alle Messungen erfolgten bei maximaler Visusforderung in 5 m. Die Befunde unter binokularen Sehbedingungen wurden ausgewertet. Der Prismeneffekt der Brillengläser [10], der evtl. eine Differenz zwischen der KZH und Position der Neutralzone bedingen könnte, betrug nur in 2 Fällen >5° (Tab. 1, Nr. 7, 27). Er ist in der Auswertung vernachlässigt.

**Table 1:** 

Nr	A/G	Diagnose Auffälligkeit	Refraktion in Zykloplegie		BL (mm)	Dosis mm/Auge	KZH Ferne (Grad)		KZH Nähe (Grad)		Motilität (mm)		Binokularer Visus		Binokularfunktion		NBZ (Mon.)
			RA	LA			Prä	Post	Prä	Post	Ad	Ab	Prä	Post	Prä	Post	
1	20 w	Fovea-OCT	−3,5/155°	−1,0/−2,75/25°	24,2	10	25 L	20 L	0	0	7	6	0,12	0,12	1	1	27
2	10 m	Albinismus	−3,5/3,0/16°	−2,5/−4,75/160°	25,8	12	30 L	20 L	30 L	15 L	7	7	0,5	0,5	4	3	30
3	12 m	Idiopathisch	+2,0/−4,0/7°	+1,0/−1,25/175°	24,0	12	30 R	20 R	15 R	5 R	6	6	0,63	0,5	4	4	23
4	6 w	Idiopathisch	−0,5/−1,25/116°	+0,25/−2,75/20°	21,4	10	30 L	20 L	20 L	12 L	7	7	0,8	1,0	4	4	8
5	7 m	Fovea-OCT	−3,25/−3,75/25°	−3,5/−3,25/158°	24,6	12	20 L	0	20 L	0	6	6	0,2	0,32	2	2	19
6	4 m	Albinismus	+2,25/−0,75/152°	+3,5/−1,0/174°	21,2	12	35 L	15 L	30 L	10 L	5	5	0,5	0,8	4	4	20
7	5 w	Idiopathisch	+4,75/−1,25/169°	+5,25/−2,5/177°	21,6	10	40 L	17 L	20 L	10 L	6	6	0,63	0,63	4	4	43
8	15 w	Idiopathisch	−1,5/−4,25/6°	−1,25/−4,75/172°	24,2	13	30 L	10 L	20 L	10 L	2	6	0,5	0,4	1	0	12
9	6 w	Albinismus	+5,25/−5,5/11°	+4,5/−3,75/160°	21,6	10	20 R	5 R	5 R	0	6	9	0,4	0,4	2	3	45
10	6 m	Idiopathisch	+5,25/−1,75/110°	+6,75/−2,0/54°	21,6	16^a^	40 R	3 R	30 R	3 R	4	4	0,5	0,8	4	4	8
11	44 w	NF 1	−0,25/−0,25/150°	+0,25 sph	23,8	14	35 R	15 R	15 R	10 R	6	7	1,0	0,8	4	4	22
12	6 m	Idiopathisch	+4,25/1,5/3°	+2,75/−0,5/161°	22,5	14	30 R	20 R	30 R	10 R	7	6	1,0	1,0	4	4	23
13	8 m	Fovea-OCT	+5,75/−1,75/172°	+5,75/−1,25/1°	21,3	13	30 L	10 L	25 L	0	–	–	0,8	0,8	4	4	23
14	9 m	Frühgeburt	+1,75/−1,0/176°	+0,5/−1,25/136°	21,7	13	30 L	5 L	25 L	5 L	6	6	1,0	1,0	4	4	12
15	6 m	Fovea-OCT	+6,5/−1,75/159°	+7,0/−1,5/174°	21,4	13	35 R	15 R	35 R	10 R	6,5	6,5	0,5	1,0	4	4	20
16	8 w	Idiopathisch	+0,75/−0,25/25°	+0,75/−0,5/61°	22,9	13	40 R	12 R	20 R	0	6	7	1,0	1,0	4	4	13
17	8 m	Idiopathisch	+1,0/−1,0/39°	+1,25/−0,25/178°	22,8	13	40 L	15 L	20 L	10 L	7	6	0,63	0,63	4	4	13
18	8 m	Idiopathisch	+3,25 sph	+3,0/−0,25/0°	22,3	13	40 L	5 L	10 L	0	5,5	6	1,0	1,25	4	4	11
19	7 m	Idiopathisch	+2,25/−0,75/168°	+1,5/−0,5/174°	21,7	14	30 L	10 L	30 L	10 L	6	6	0,8	0,63	3	2	12
20	34 m	Idiopathisch	+0,5/−1,0/9°	+0,75/−1,0/1°	23,6	14	35 L	8 L	20 L	4 L	7,5	8	1,0	1,0	4	4	12
21	5 w	Idiopathisch	+1,0/−2,0/9°	+1,0/−2,0/159°	–	12	35 L	15 L	30 L	10 L	6	7	0,4	0,63	4	4	27
22	6 m	Idiopathisch	+0,25/−0,5/86°	−0,25 sph	23,4	14	40 L	10 L	10 L	5 L	5	6	0,8	1,0	4	4	14
23	7 m	Retardierung	+2,0/−1,5/7°	+2,75/−2,25/170°	22,4	14	35 L	3 L	25 L	0	≥2	≥3	0,5	0,4	3	4	15
24	7 m	Fovea-OCT	+4,5/−1,5/2°	+5,5/−1,5/169°	20,3	10,5^b^	30 R	12 R	20 R	7 R	6	5,5	0,5	0,4	1	3	15
25	6 m	Fovea-OCT	+2,25/−1,25/10°	+1,0/−1,5/120°	21,8	13	25 L	10 L	15 L	5 L	7	7	0,4	0,5	4	4	14
26	38 w	Idiopathisch	+0,5/−0,75/173°	+0,25/−0,25/8°	22,3	10	25 L	0	10 L	0	7	6	1,0	1,0	4	4	61
27	4 m	Idiopathisch	+7,75/−0,75/20°	+8,0/−0,5/15°	19,8	14	40 L	0	40 L	12 R^c^	4	5	0,63	0,8	4	4	12
28	9 m	Optikusatrophie	−4,75/−0,25/70°	−5,5/−1,0/170°	25,2	13	25 L	2 L	10 L	0	6	6	0,4	0,32	3	3	12
29	7 w	Albinismus	+1,75 sph	+2,0/−0,75/168°	21,2	14	40 L	15 L	40 L	0	4	6	0,5	0,8	1	1	12
30	11 m	Fovea-OCT	+2,25/−0,5/10°	+2,0 sph	23,6	14	30 L	5 L	10 L	2 L	6	6	1,0	1,0	4	4	12
31	7 m	Idiopathisch	+3,5/−0,75/16°	+3,5/−0,75/8°	21,8	14	35 R	20 R	25 R	15 R	4	4	0,8	1,25	4	4	8
32	12 m	Albinismus	−1,25/−1,75/25°	−1,25/−2,0/155°	24,0	14	25 L	5 L	15 L	7 L	6	7	0,2	0,2	3	3	12
33	6 m	Nachtblindheit	+3,25/−0,5/38°	+4,0−0,75/75°	22,1	14	45 R	0	40 R	–	5	5	0,5	0,5	4	4	14

Idiopathisch: Fälle mit normalem Makula-OCT (kein ERG), Fovea-OCT: subdifferenzierte Fovea, keine sonstige Auffälligkeit

*A* Alter/Jahre, *G* Geschlecht, *BL* axiale Bulbuslänge, Mittelwert beider Augen; *KZH* Kopfdrehung (Grad), *NBZ* Nachbeobachtungszeit, *prä* präoperativ, *post* postoperativ, *R* rechts, *L* links

Binokularfunktion: 1 = Bagolini, 2 = Titmus-Fliege, 3 = Titmus-Ringe, 4 = Lang-Stereotest

^a^15/17, geringere Dosis medial, jeweils 8 mm Verlängerung mit bovinem Perikard-Interponat

^b^9/12, nach [11]

^c^Umkehr der KZH bei dekompensierter Exophorie

### Operation

Alle Eingriffe erfolgten in Allgemeinanästhesie. Über einen Limbusschnitt wurde der Muskel nach Lösen vom umgebenden Bindegewebe bis zur Tenonpforte am oberen und unteren Sehnenviertel mit Polyglactin 6‑0 angeschlungen. Die Rücklagerungsstrecken wurden von 2 Referenzpunkten am Limbus aus gemessen. Bei den AO wurden beide Muskeln in der Regel um den gleichen Betrag rückgelagert. Die KO erfolgten mit Verkürzung durch Faltung, selten durch Resektion. Dabei wurden jeweils das obere und untere Sehnendrittel mit Polyglactin 6‑0 angeschlungen und am Ober- und Unterrand der Insertion skleral fixiert. Auch bei den KO war die Dosisverteilung symmetrisch.

### Statistik

Die Mediane und Streubreiten für Alter, Operationsdosis, prä- und postoperative KZH sowie den prä- und postoperativen Binokularvisus wurden ermittelt. Als Erfolgsraten wurden der Anteil auf ≤10° und ≤15° Rest-KZH korrigierter Patienten definiert. Zum Vergleich der Gruppen AO und KO diente der Mann-Whitney-U-Test. Konfidenzintervalle wurden nach der Formel von Hald berechnet. Die Untersuchung der Geschlechts- und Seitenwendigkeit erfolgte mit der Binomialfunktion. Zur Berechnung der Visusmittelwerte wurden die Werte logarithmiert und das arithmetische Mittel der logarithmierten Werte in den Visus zurücktransformiert.

## Ergebnisse

**Figure 1:**
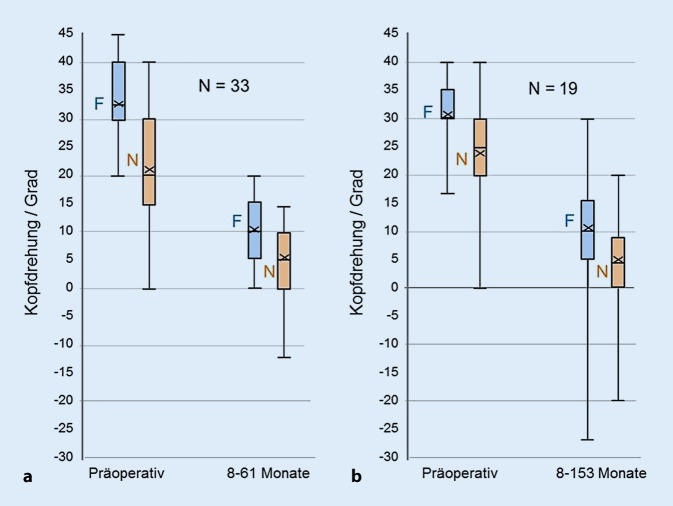

Wesentliche Patientendaten sind in Tab. 1 und 2 zusammengefasst. Es überwogen 37 (71 %) männliche gegenüber 15 (29 %) weiblichen Patienten. Linksdrehung war mit 71 %, Rechtsdrehung mit 29 % vertreten (*p* = 0,0016). Die Altersverteilung und die Größe der KZH in den Gruppen waren sehr ähnlich (Abb. 1). Der Altersmedian lag bei 7 (AO) bzw. 6 (KO) Jahren.

**Table 2:** 

Nr	A, G	Diagnose Auffälligkeit	Refraktion in Zykloplegie		BL (mm)	Dosis/Auge (mm)	KZH Ferne (Grad)		KZH Nähe (Grad)		Motilität (mm)		Binokularer Visus		Binokularfunktion		NBZ (Mon.)
			RA	LA			Prä	Post	Prä	Post	Ad	Ab	Prä	Post	Prä	Post	
1	15 m	Albinismus	−1,25 sph	−1,0/−0,5/40°	23,5	20	30 L	10 L	20 L	5 L	40	35	0,8	0,8	4	4	55
2	11 m	Idiopathisch	+2,0/−0,25/0°	+2,0/−0,5/165°	22,5	17	17 L	14 L	0	5 L	40	30	1,0	0,8	4	4	64
3	5 m	Albinismus	+2,5/−0,5/11°	+2,0/−0,25/1°	22,0	12	30 L	15 L	30 L	8 L	32	35	1,0	0,8	4	4	29
4	12 m	Idiopathisch	+2,5/−2,75/164°	+1,5/−0,75/175°	–	15	25 R	7,5 R	10 R	0 R	30	40	1,0	1,0	3	3	19
5	6 w	Idiopathisch	+4,0/−2,0/169°	+4,5/−2,25/4°	22,8	19	30 L	25 L	20 L	20 L	35	32	0,8	1,25	4	4	38
6	6 m	Optikusatrophie	+0,25/−0,75/171°	+0,5/−0,25/80°	22,2	20	30 L	0	20 L	0	30	40	0,8	0,63	0	0	92
7	6 m	Idiopathisch	+2,25 sph	+2,0 sph	23,6	24	40 L	5 L	37 L	2 L	45	40	0,8	1,0	4	4	107
8	18 m	Idiopathisch	+3,0 sph	+4,0/−2,0/39°	22,7	20	35 L	0	35 L	0	25	30	0,5	0,63	3	3	15
9	7 m	Idiopathisch	+1,75/−1,75/3°	+1,5/−1,0/15°	22,9	20	35 R	0	5 R	0	25	20	1,0	0,8	4	4	63
10	4 m	Idiopathisch	+2,0/−0,75/129°	+1,25/−0,5/73°	22,5	20	35 L	10 L	35 L	15 L	27	27	0,8	0,8	2	2	15
11	4 w	Idiopathisch	+2,25/−0,75/162°	+1,5/−0,75/61°	21,5	20	40 R	15 R	30 R	5 R	30	30	0,8	1,0	4	3	90
12	8 m	Albinismus	+5,0/−0,5/30°	+4,5/−0,5/125°	21,8	24	30 L	7 L	25 L	3 L	35	40	0,5	1,0	1	1	140
13	5 m	Optikusatrophie	+1,25/−1,25/30°	+0,5/−1,5/180°	23,3	24	40 L	10 L	40 L	15 L	27	30	0,2	0,32	1	1	18
14	27 w	Idiopathisch	+1,0/−0,25/165°	+1,25/−0,5/165°	22,7	20	35 L	7 L	10 L	5 R	25	25	1,0	1,25	3	3	32
15	6 w	Albinismus	+3,25/−0,75/54°	+4,0 sph	22,1	13	35 R	30 R	15 R	10 R	45	40	0,8	1,0	4	4	153
16	7 m	Idiopathisch	+5,0/−0,5/0°	+4,5/−1,0/10°	22,5	18	30 L	17 L	30 L	7 L	40	40	0,5	0,63	4	4	20
17	8 m	Idiopathisch	+1,5/−0,5/170°	+1,5/−0,5/30°	21,9	20	30 R	27 L^a^	25 R	20 L^a^	12	10	1,0	1,0	4	3	84
18	6 w	Idiopathisch	+3,75/−1,25/160°	+3,75/−0,5/159°	21,6	18	32 R	15 R	27 R	0	30	25	0,5	0,8	3	3	34
19	6 m	Idiopathisch	+2,5/−1,5/135°	+2,0 sph	22,5	18	30 L	10 L	30 L	12 L	40	40	0,8	0,8	4	4	35

Idiopathisch: Fälle ohne OCT oder mit normalem Makula-OCT (kein ERG); *A* Alter, *G* Geschlecht, *BL* axiale Bulbuslänge, Mittelwert beider Augen, *KZH* Kopfdrehung (Grad), *NBZ* Nachbeobachtungszeit, *prä* präoperativ, *post* postoperativ, *R* rechts, *L* links

Binokularfunktion: 1 = Bagolini, 2 = Titmus-Fliege, 3 = Titmus-Ringe, 4 = Lang-Stereotest

^a^Spätere Umkehr der KZH

Die KZH betrug im Median 32,5° (AO) bzw. 30° (KO), die Rücklagerungsstrecke im Rahmen der AO 13 mm (Median), zu Beginn 10–12 mm (*n* = 10), später ≥13 mm (*n* = 23). Die KO war KZH-abhängig mit einer mittleren Gesamtdosis von 0,60 mm/Auge/Grad Kopfdrehung ca. 10 % höher dosiert als in einer früheren Studie aus dieser Klinik [6]. Die Operationszeit der AO war im Vergleich zur KO 40 % kürzer. Die Tab. 3 zeigt im Vergleich die KZH präoperativ und bei den postoperativen Kontrollen. Die KZH wurde durch AO längerfristig um 67 %, durch KO um 64 % reduziert, bei Beschränkung auf die 9 Patienten mit Spätkontrolle <3 Jahre nach KO um 70 % (43–100). Diesbezüglich bestand kein signifikanter Unterschied zwischen den Verfahren (*p* = 0,83) Eine erfolgreiche Korrektur mit Restdrehung ≤10° wiesen zur Spätkontrolle nach AO 55 % der Patienten auf, nach KO 57 % und 65 % nach AO ≥13 mm. Im Bereich ≤15° lagen nach AO 79 %, nach KO 81 % und 91 % nach AO ≥13 mm (*n* = 23).

**Table 3:** 

	Anderson-Operation (*n* = 33)	Kestenbaum-Operation (*n* = 19)
Zeitraum der Operationen	9/2013–6/2018	4/2003–4/2013
Alter bei Operation/Jahre	7 (4–44)	6 (4–27)
KZH Ferne präoperativ/°	32,5 (20–45)	30 (17–40)
OP-Strecke pro Auge/mm	13 (10–16)	10 (6–12) + 10 (6–12)
KZH Ferne nach 3 bis 4 Monaten/°	10 (−3–20)	10 (−3–20)
KZH-Reduktion/°	25 (10–38)	20 (7–38)
KZH-Reduktion/%	75 (33–109)	70 (33–109)
Ab‑/Adduktion/°	30/30 (15–35/20–35)	35/30 (12–40/12–40)
Nachbeobachtung/Monate	14 (8–61)	36 (15–153) (*n* = 18)
KZH Ferne spät/°	10 (0–20)	10 (0–30) (*n* = 18)
KZH-Reduktion/°	20 (5–37)	21 (3–35) (*n* = 18)
KZH-Reduktion/%	67 (20–100)	64 (14–100) (*n* = 18)
Ab‑/Adduktion/°	30/30 (10–37/15–45)	32/30 (10–40/12-45) (*n* = 18)

*KZH* Kopfzwangshaltung; hier Kopfdrehung

Die KZH bei Nahfixation war deutlich geringer als in der Ferne. Sie betrug in der AO-Gruppe 20° (0–40), bei der Spätkontrolle 5° (0–15), lediglich ein hoch hyperopes Kind (Tab. 1, Nr. 27) mit 19,8 mm Bulbuslänge, bei dem der Eingriff eine Exophorie induzierte, drehte in der Nähe zur Gegenseite. In der Gruppe KO wurde die Kopfdrehung bei Nahfixation von 25° (0–40) auf 4° (0–20) reduziert (Abb. 1).

Elf Patienten wiesen vor AO eine Kopfneigung ≥10° (10–30) auf. Sie war zur Spätkontrolle in 2 Fällen 10° geringer, in 1 Fall 10° größer, in den übrigen Fällen weitgehend unverändert (Differenz 0–5°). Vor KO hatten 4 Patienten eine Kopfneigung ≥10° (10–15). Sie war zur Spätkontrolle in 3 Fällen 10° geringer, in 1 Fall 7° größer.

Der mittlere Binokularvisus betrug vor/nach AO im Mittel 0,58/0,63, vor/nach KO im Mittel 0,72/0,82 (logMAR 0,24 ± 0,22/0,20 ± 0,23 bzw. logMAR 0,14 ± 0,17/0,084 ± 0,13). Der mittlere Anstieg betrug somit ca. 1/2 Zeile. In der Altersklasse ≤6 Jahre (*n* = 10) entsprach die Verbesserung von logMAR 0,24 ± 0,13 auf logMAR 0,14 ± 0,14 nach AO 1 Zeile. In der Altersklasse >6 Jahre blieb der mittlere Visus von 0,58 unverändert.

## Diskussion

Die Ergebnisse dieser Studie bestätigen die Wirksamkeit beider Verfahren. Beide, AO und KO reduzierten die KZH deutlich, behoben sie aber in der Regel auf Dauer nicht komplett. Die hoch dosierte AO und die invasivere KO mit der fast doppelten, auf 4 Muskeln verteilten Gesamtdosis waren gleichwertig. Der Vergleich mit den KO erscheint aufgrund der nur kleinen Änderung im späteren Verlauf trotz unterschiedlicher Nachbeobachtungszeit möglich. Die präoperative KZH war sehr ähnlich aufgrund der ausschließlichen Anwendung der AO bzw. KO in den beiden Zeiträumen. Für das Kriterium Restdrehung ≤15° lagen die Erfolgsraten bei 79 % (AO) und 81 % (KO), für das Kriterium KZH ≤10° bei 55 % (AO) bzw. 57 % (KO). Nach AO mit ≥13 mm Rücklagerung erzielten 91 % eine KZH ≤15° und 65 % eine KZH ≤10°. Das entspricht bezüglich der KO den Ergebnissen einer früheren Studie aus unserer Klinik [6]. Das Gros der Patienten war wiederum männlich, damals mit 64 %, nun mit 71 %. Die Linksdrehung überwog mit damals 65 %, nun 71 %, bei männlichen Patienten mit ca. 3:1 (*p* = 0,0057), bei weiblichen mit ca. 3:2 (*p* = 0,12).

Die KZH bei Nahfixation ist oft geringer, mitunter wesentlich geringer als bei Fixation in der Ferne, selbst wenn Konvergenz fordernde Prismen bei Fernfixation keine Änderung der KZH bewirken oder die nötige Fusionsbreite fehlt. Es wurde gewarnt, dass eine Dosiserhöhung der KO mit einer Überkorrektion bei Nahfixation einhergehen könne [13]. Uns sind diesbezüglich keine systematischen Studien bekannt. Nach den Ergebnissen unserer Studie, in der vielleicht erstmalig die KZH differenziert auch für die Nähe analysiert wurde, ist eine Überkorrektion für die Nähe nicht zu befürchten. So waren z. B. die Patienten 2, 9, 14 in der Gruppe KO (Tab. 2) sowie 1, 18, 22, 30 in der Gruppe AO (Tab. 1) mit deutlich geringerer KZH bei Nahfixation trotz hoher Dosis nicht überkorrigiert. Die einzige Überkorrektion für die Nähe (Tab. 1, Nr. 27) war durch Dekompensation einer Exophorie bedingt, bei präoperativ gleich großer KZH in Ferne und Nähe.

Viele Autoren bevorzugen asymmetrische Dosisverteilungen mit größeren Strecken lateral als medial, besonders bei der AO. Die von uns bevorzugte gleiche Verteilung könnte wegen der medial relativ zu lateral kürzeren Muskelabrollstrecke und dadurch medial stärkeren Reduktion des muskulären Drehmoments eine Exostellung induzieren. Außer bei dem hoch hyperopen Kind (Tab. 1, Nr. 27) mit entsprechend kleinen Augen (das wegen der großen KZH von 40° dennoch Rücklagerungen von 14 mm erhielt) wurde jedoch selbst unmittelbar nach der Verbandabnahme keine Exophorie beobachtet. Bei den wenigen Reoperationen fiel auf, dass die Muskeln 2–3 mm vor dem Fixationsort des ersten Eingriffs inserierten [7]. Als Ursache vermuten wir eine Faltenbildung der Sehne durch einen nach vorn gerichteten Schub des Bindegewebes bzw. der Tenonpforte. Ob die effektive Rücklagerung immer der applizierten Dosis gleichkommt, ist daher nicht garantiert.

In der Literatur sind auch nach niedriger dosierter AO bemerkenswerte Effekte beschrieben. Nicht selten war die KZH mit 45° angegeben, mehr als wir in der Regel fanden [28]. Nicht selten wurde trotz geringerer Rücklagerungsstrecken Vollkorrektur beschrieben, was in unserer Studie ebenfalls die Ausnahme war. Eine Übersicht gibt die Tab. 4. Kopfdrehungen von im Median 35° (15–45) wurden demnach durch Rücklagerungen von medial 4–8 mm und lateral 6–12 mm stark reduziert oder komplett behoben [17]. Verglichen mit den eigenen Ergebnissen sind diese Effekte erstaunlich. In einer anderen Studie wurden Kopfdrehungen von 40° durch Rücklagerungen entsprechend medial 8 mm und lateral 7 mm auf 10° verringert. Es resultierten sogar Übereffekte [2]. Einige Patienten schielten allerdings manifest und die Dosis war entsprechend modifiziert [2]. Nach Schielkorrektur kann auch Binokularsehen den Nystagmus dämpfen, und eine Umkehr der Kopfdrehung kann bei Vorliegen eines Latenstypnystagmus durch den Wechsel der Fixation aufs andere Auge bedingt sein. Die Ergebnisse kombinierter Nystagmus- und Schielchirurgie sind daher zur Beurteilung des reinen Umlagerungseffekts ungeeignet. In der Studie aus Indien an 13 orthotropen Patienten wurde die KZH von 30° durch 9 mm Medialis- und 12 mm Lateralisrücklagerung bei einer Nachbeobachtungszeit von 3 Monaten gut reduziert, nur in wenigen Fällen war die Wirkung schwach [11]. Die Abnahme der mittleren Rest-KZH um 2° in den ersten 3 Monaten war nicht signifikant [11]. In unserer Studie nahm die Rest-KZH zwischen dieser Kontrolle nach 3 Monaten und der Spätkontrolle nach AO und KO im Mittel um ca. 2° zu. In einer Studie aus Südkorea erzeugten asymmetrische Rücklagerungen von 10–14 mm in 2 von 13 Fällen einen Übereffekt von 10° [29]. Die KZH betrug dabei präoperativ maximal 30° [29]. Auch in einer früheren Studie erfolgte die Indikation zur AO vorzugsweise bei geringerer KZH [17].

**Table 4:** 

Autoren/Studie	*N* Pat	Dosis m/l (mm/Auge)	KZH Median (min–max)	Rest-KZH ≤10° Prozentanteil (95 % KI)	Rest-KZH ≤15° Prozentanteil (95 % KI)	NBZ (Mon.)
Gupta et al. [11]	12	9/12	30 (25–40)	75 (43–93)	92 (60–99)	3
Garcia-Guzman et al. [5]	10	4–8/6–12	30 (15–45)	70 (35–92)	100 (66–99)	6–24
Teodorescu et al. [26]	15	7–9/10–11,5	30 (25–40)	100 (75–99)	100 (75–99)	6–48
Kumar et al. [17]	8	6–11,5/9–13,5	25 (15–30)	88 (47–99)	88 (47–99)	6–13
Yang et al. [29]	13	10/12	20 (15–30)	92 (62–100)	100 (72–99)	6–183
Yahalom et al. [28]	27	10/12	40 (25–45)	52 (32–71)	89 (70–97)	6–108
Aktuelle Studie gesamt	33	9–15/10–17	35 (20–40)	55 (37–71)	79 (61–90)	8–43
Aktuelle Studie ≥13 mm	23	13–15/13–17	35 (30–40)	65 (43–83)	91 (70–98)	8–23

*KZH* Kopfdrehung in Grad, *m/l* medial/lateral, *KI* Konfidenzintervall, *NBZ* Nachbeobachtungszeit, *Mon.* Monate

Ein schwacher Effekt kann durch sparsame Muskelpräparation entstehen, auch durch eine zu niedrige Dosis, wenn der Patient z. B. die maximale bzw. der Lage der Ruhezone entsprechende Kopfdrehung wegen des dafür zu kleinen Brillengestells oder optischer Nebenwirkungen der Gläser nicht einnimmt. Ein falsch zu hoher Operationseffekt ergibt sich, wenn die KZH zwar präoperativ, aber nicht auch postoperativ unter maximaler Visusforderung bestimmt wird oder die KZH präoperativ überschätzt wurde. Bei 45° Kopfdrehung stellt sich die Frage, ob eine weit laterale Ruhezone vorliegt oder ob erst maximale Blickwendung den Nystagmus hemmt, erst recht in Fällen, bei denen eine Drehung von erstaunlichen 50° angegeben wird [28]. In diesem Fall der Beruhigung durch den seitlichen Anschlag ist eine Vollkorrektur der KZH trotz noch vorhandener Restmotilität schwer zu verstehen. Eine Fehleinschätzung der Kopfdrehung durch bloßes Schätzen, bei unruhigen Patienten auch mit dem Goniometer, kann mit der von uns beschriebenen Messmethode an einer Tangentenskala vermieden werden.

Es wurde wiederholt über Visusverbesserungen nach Umlagerungsoperationen berichtet. Wir fanden weder nach KO noch AO einen wesentlichen Visusanstieg. Der Anstieg war ähnlich gering wie in einer aktuellen Studie [29]. Die Differenz von ca. 1/2 Stufe könnte aus der etwas besseren Korrektionswirkung der Brille resultieren, weil der Durchblick postoperativ näher am optischen Zentrum der Gläser erfolgt. Es fiel jedoch kein Zusammenhang mit der Höhe der Ametropie auf, der diese Erklärung stützen könnte. Bei Nachkontrollen von Kindern ist zu bedenken, dass in diesem Alter noch Visusreifung stattfindet. Die Befunde in der Gruppe AO deuten auf einen Einfluss dieses Faktors hin. In der Altersklasse bis zu 6 Jahren betrug der mittlere Visusanstieg 1 Zeile, in der Altersklasse ab 7 Jahren war der Visus postoperativ unverändert. Der Benefit der operativen Behandlung bestand in der Reduktion der KZH.

## Fazit für die Praxis

Hoch dosierte Anderson-Operationen und Kestenbaum-Operationen waren bezüglich der Reduktion der Kopfzwangshaltung gleichwertig. Die hoch dosierte Anderson-Operation ist weniger invasiv, und die Operationszeit ist kürzer. Sie ist damit eine sehr gute Alternative zur Kestenbaum-Operation. Seitengleiche Rücklagerungen der agonistischen Horizontalmotoren mit einer Standarddosis von 13–14 mm sind zur Korrektur von Kopfdrehungen zwischen 25 und 45° geeignet.
